# Fatigue Characterization of EEG Brain Networks Under Mixed Reality Stereo Vision

**DOI:** 10.3390/brainsci14111126

**Published:** 2024-11-07

**Authors:** Yan Wu, Chunguang Tao, Qi Li

**Affiliations:** 1 School of Computer Science and Technology, Changchun University of Science and Technology, Changchun 130022, China; wuyan@cust.edu.cn (Y.W.); 2022100987@mails.cust.edu.cn (C.T.); 2 Jilin Provincial International Joint Research Center of Brain Informatics and Intelligence Science, Changchun 130022, China; 3 Laboratory of Brain Information and Neural Rehabilitation Engineering, Zhongshan Research Institute, Changchun University of Science and Technology, Zhongshan 528437, China

**Keywords:** mixed reality (MR), electroencephalography (EEG), visual fatigue, brain network, phase-locked value (PLV)

## Abstract

Mixed Reality (MR) technology possesses profound and extensive potential across a multitude of domains, including, but not limited to industry, healthcare, and education. However, prolonged use of MR devices to watch stereoscopic content may lead to visual fatigue. Since visual fatigue involves multiple brain regions, our study aims to explore the topological characteristics of brain networks derived from electroencephalogram (EEG) data. Because the Phase-Locked Value (PLV) is capable of effectively measuring the phase synchronization relationship between brain regions, it was calculated between all pairs of channels in both comfort and fatigue states. Subsequently, a sparse brain network was constructed based on PLV by applying an appropriate threshold. The node properties (betweenness centrality, clustering coefficient, node efficiency) and edge properties (characteristic path length) were calculated based on the corresponding brain network within specific frequency bands for both comfort and fatigue states. In analyzing the PLV of brain connectivity in comfort and fatigue states, a notable enhancement in brain connectivity is observed within the alpha, theta, and delta frequency bands during fatigue status. By analyzing the node and edge properties of brain networks, it is evident that the mean values of these properties in the fatigue state were higher than those in the comfort state. By analyzing the node and edge properties at a local level, the average difference in betweenness centrality, clustering coefficients, and nodal efficiency across the three EEG frequency bands was computed to find significant brain regions. The main findings are as follows: Betweenness centrality primarily differs in frontal and parietal regions, with minor involvement in temporal and central regions. The clustering Coefficient mainly varies in the frontal region, with slight differences being seen in the temporal and occipital regions. Nodal efficiency primarily varies in the frontal, temporal, and central regions, with minor differences being seen in the parietal and occipital regions. Edge property analysis indicates that there is a higher occurrence of long-distance connections among brain regions during the fatigue state, which reflects a loss of synaptic transmission efficiency on a global level. Our study plays a crucial role in understanding the neural mechanisms underlying visual fatigue, potentially providing insights that could be applied to high-demand cognitive fields where prolonged use of MR devices leads to visual fatigue.

## 1. Introduction

Head-Mounted Displays (HMDs) support the application of virtual reality (VR), augmented reality (AR), and mixed reality (MR) technologies across diverse fields, offering users rich and immersive visual experiences [1]. The advancements and growing popularity of HMDs have driven the widespread application of those technologies across various new fields [2,3,4]. However, as the eyes constantly shift focus and converge to track moving objects, some studies on HMDs suggest that prolonged visual experiences may elicit adverse reactions, including ocular fatigue, vertigo, and a range of visual discomforts [5,6,7]. Consequently, in recent years, the fatigue characteristic associated with HMDs has garnered significant attention. The use of eye movement parameters [8,9] as means to assess visual fatigue induced by virtual reality (VR) and augmented reality (AR) technologies has become relatively mature. Currently, the assessment of fatigue resulting from MR technology frequently employs subjective questionnaires [10]. Nevertheless, subjective assessments are vulnerable to variations among individuals, and there is no uniformity in the evaluation standards [11]. Neural network theory emerges as an objective means to evaluate fatigue [12,13], aiming to compensate for the deficiencies inherent in subjective evaluations. It remains unclear whether visual stimuli in MR environments involve changes in multiple brain regions. The specific effects and mechanisms are not yet fully understood and require further investigation.

Recently, non-invasive electroencephalogram (EEG) signals have surfaced as a promising tool for the real-time monitoring of brain information streams [14], which consistently indicate the extent of visual fatigue [15,16,17]. However, the information derived from individual EEG channels did not directly reflect the global inter-channel correlations. EEG-based functional brain connectivity (FBC) has the potential to uncover the neural mechanisms underlying neurophysiological disorders [18], which can be utilized to investigate the changes in brain regions when a state of visual fatigue occurs. Wang et al. [19] concluded that the P270 component elicited by 3D stereo stimuli might indicate functional alterations in the occipital regions. Chen et al. [20] revealed differences in brain activation and discovered that the frontal, temporal, central, and parietal regions exhibited heightened activation when employing functional magnetic resonance imaging (fMRI) to investigate visual fatigue induced by viewing 3D televisions. Yue and Wang discovered that, as participants experienced fatigue, there was a significant reduction in event-related spectral perturbation (ERSP) within the theta band located in the central-frontal area, with a latency ranging from 200 to 300 milliseconds [21]. The distribution of brain regions with key connections is revealed, facilitating the establishment of brain network characteristics. It is possible that the development and effects of visual fatigue caused by MR visual stimuli involve multiple brain regions.

As different frequency bands correspond to unique brain network characterizations, it is essential to investigate these characteristics through the analysis of different frequency bands. In spatial tasks, the delta, alpha, and theta frequency bands are the most widely researched in terms of brainwave activity, showing significant variations and specific patterns in visual experiences [22]. Wang et al. [19] discovered that the alpha, theta, and delta frequency bands were predominant during the calculation of the topography of directed transfer function (DTF) information flow under various parallax conditions. Zhang et al. explored VR-induced fatigue by analyzing brain networks and found increased fatigue in brain network parameters within the alpha and theta frequency bands [23]. Han et al. found that the delta rhythm manifested heightened functional connectivity and larger clustering coefficients as fatigue intensified, concurrently leading to a reduction in the average shortest path length [24]. Yu et al. investigated the neural mechanisms by comparing brain networks between the 3D and 2D groups. They discovered that brain networks for beta and gamma waves had higher global efficiency in the 3D group; however, the brain networks linked to alpha waves in the 2D group had higher frontal efficiency [25].

To investigate multiple brain regions involved in MR visual stimuli, an experiment paradigm was designed to induce visual fatigue. It has been found that, with the rise in fatigue, there is a corresponding increase in the synchrony between different brain regions [26]. The properties of brain network topology, which are associated with functional brain connectivity, can be regarded as significant indicators for assessing fatigue [23]. In this paper, the phase-locked values (PLVs) were utilized as connectivity metrics to capture temporal correlations between brain signals. Utilizing this approach, a brain network was constructed based on PLVs. The topological properties of this network were adopted as measures for the neural network analysis. This study systematically compared brain network characteristics across the alpha, theta, and delta frequency bands in comfort and fatigue states and discussed the underlying neural mechanisms between brain regions.

## 2. Materials and Methods

### 2.1. Preparatory Experiment

**Figure 1:**
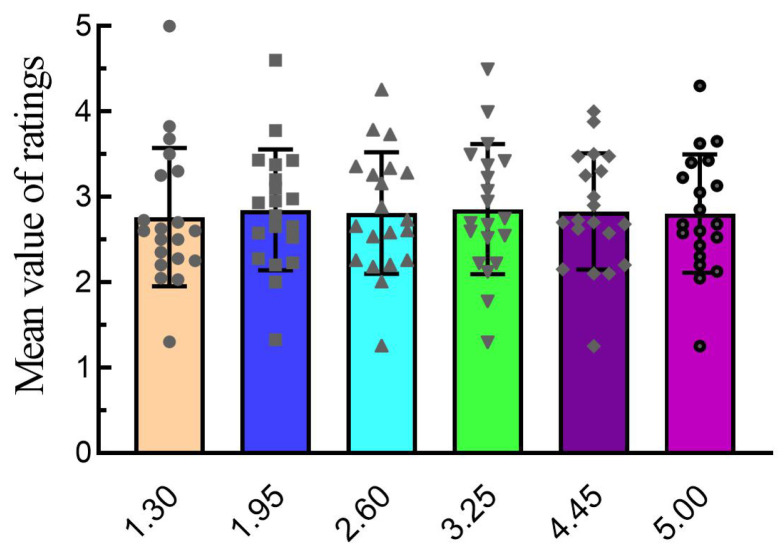
Average ratings for the six-speed modes.

Since each participant had different sensitivities to fatigue that were triggered by different speeds, we invited 20 participants to watch a preparatory experimental paradigm with six different speeds, and the participants subjectively rated their state of fatigue after watching each speed.Participants must calibrate their eye-tracking on the Microsoft HoloLens2 upon first use to verify normal stereo vision. They also need adequate sleep prior to the experiment to prevent eye fatigue. The preliminary experiment comprised 20 blocks. Each block included preparation scenes, movement scenes, rating scenes, and an end scene. Each type of scene required participants to maintain their gaze for a specified duration before proceeding to the next scene in order. Among them, preparation scenes aim to attract the participants’ attention. In movement scenes, a green gem moved in periodic uniform motions at one of six distinct speeds. The rating scenes encompass an evaluation of the eye’s present dryness and pleasant levels, with each level rated on a scale of 1 to 5, where lower scores correspond to fatigue and higher scores correspond to comfort. The average of these two levels’ ratings is calculated to assess each speed. Ratings were made on a five-point scale, with low to high scores ranging from fatigue to comfort. A single block cycled six trails. Across 20 blocks, this resulted in a total of 120 trails. All participants’ scores for each speed mode were statistically analyzed. The outcomes of the statistical analysis are depicted in Figure 1. There were no significant differences in ratings for all six-speed modes. The stereo depth motion, with a speed of 3.25 m/s, was finally chosen for EEG experiments.

### 2.2. EEG Experiments

**Figure 2:**
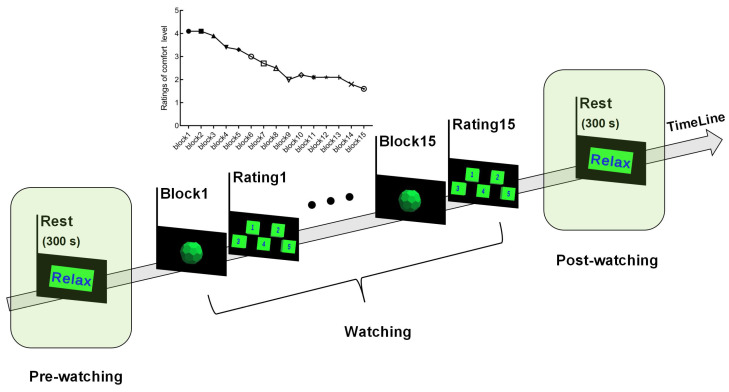
Flow diagram of EEG experiments.

Eighteen healthy participants from the preparatory experiment (mean age: 24.17 ± 1.30, including 13 males and 5 females) were invited to participate in EEG experiments. The Microsoft HoloLens2 device retained eye-tracking calibration data for all participants. Before the experiment, all participants were asked to sign a consent form, ensure adequate sleep the night before, and avoid alcohol, coffee, or other stimulants. The diagram illustrating the EEG experimental process is displayed in Figure 2. First, participants watched the “Relax” scene for 5 min as a benchmark for assessing “comfort”. Each block consisted of one movement scene. The motion scene was consistent across each block. Each movement scene contained 20 trials, and each trial performed a reciprocating periodic stereo-depth motion at 3.25 m/s. At the end of each block, participants rated their current comfort level on the same 5-point scale as used in the preparatory experiment. A lower score signified a higher level of fatigue. As the number of blocks increases, the score exhibits a gradual decline (Figure 2). The experiment spanned roughly 62 min, during which participants were required to gaze at the corresponding scene for a certain period. If the participant was distracted for too long, the experiment was terminated and the corresponding data were invalidated. Data collected before and after watching the entire 15 blocks were labeled “comfort” and “fatigue”, respectively, and were used for subsequent analyses.

### 2.3. EEG Recording and Experimental Environment

**Figure 3:**
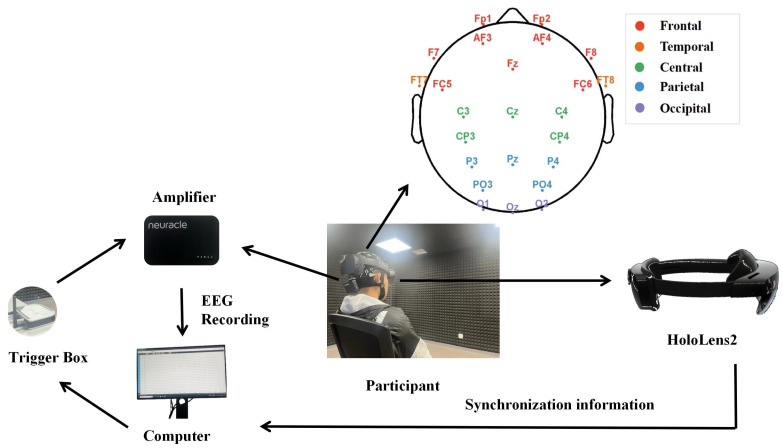
Schematic diagram of the EEG recording and experimental environment.

The NeuSen W Wireless EEG Acquisition System (developed by the Neuracle Technology Corporation, Changzhou, China) consists of a 64-electrode cap, a Neuracle amplifier, and a multi-parameter synchronizer (trigger box). The chosen electrodes exhibit a correlation with the corresponding visual brain areas as well as other regions that are implicated in the virtual environment. The brain’s occipital lobe processes visual information [27], but complex stimuli in virtual environments require the coordinated interaction of multiple brain regions [25], including the frontal, temporal, parietal, and central areas. In this study, 24 electrodes [28], each corresponding to specific brain regions, were chosen to construct the architecture of the functional brain network. These electrodes include Fp1, Fp2, AF3, AF4, F7, Fz, F8, FC5, FC6 (frontal); FT7, FT8 (temporal); C3, Cz, C4, CP3, CP4 (central); P3, Pz, P4, PO3, PO4 (parietal); and O1, Oz, O2 (occipital). The EEG signals are acquired at a sampling rate of 1000 Hz, ensuring high-resolution data capturing, while the impedance of the electrodes chosen is diligently maintained at below 10 KΩ. Figure 3 shows the experimental environment in which the EEG data were collected. The laboratory was equipped with dim light to reduce the interference of bright light in regard to the EEG signals. The background was black to help increase the contrast. Holographic lensing utilizes holographic imaging to blend the virtual scene with the real environment, providing a stimulating interface for participants and enhancing the authenticity of the mixed reality experience [29]. We used User Datagram Protocol (UDP) to accomplish synchronized signal triggering for EEG acquisition.

### 2.4. EEG Data Preprocessing

Data from eighteen participants were collected for the entire experiment. The resting-state EEG data before and after watching the 15 blocks were segmented and then preprocessed offline using the EEGLAB version 13.0.0 toolbox [30]. The EEG data were band-pass filtered (0.5–30 Hz) to remove low-frequency noise and high-frequency interference, with a notch filter at 50 Hz to eliminate powerline noise. To simplify the data analysis, we reduced the sampling rate to 250 Hz and segmented the data into 2 s epochs with no overlap. The epochs were rigorously quality-checked, with corrupted or poor-quality epochs being discarded, and any electrodes were interpolated to ensure consistency. An Independent Component Analysis (ICA) algorithm was run to isolate independent components in the data, which were then combined with the ICLABEL version 1.4 toolkit [31] to identify and remove artefacts such as Electrocardiography (ECG) and Electromyography (EMG) interference. This step further ensured the data’s quality by minimizing noise. Abnormal values were identified to remove epochs with amplitudes exceeding ±100 µV. Data from all channels were used to calculate a common average reference. In the experiment, the overall data quality remained high, with no significant impact being seen on the analysis results.

### 2.5. Brain Network Construction

After preprocessing the EEG signals, brain networks were constructed using a methodology analogous to that employed in previous studies [25,32,33]. The specific details are as follows: First, the preprocessed EEG recordings were band-pass filtered with delta (0.5–4 Hz), theta (4–8 Hz), and alpha (8–13 Hz) frequency bands. The frequency bands in question were linked to visual fatigue [11,22,23]. Second, the PLV matrix was calculated, which consisted of the functional connectivity between all possible electrode pairs. Third, the brain network was constructed based on the calculated PLV matrix by applying an appropriate threshold *T*. Finally, topological characteristics were extracted from the constructed brain networks to account for differences between comfort and fatigue states.

#### 2.5.1. Phase-Locking Value

Long-term synchronization of neural activity between all possible electrode pairs was assessed using PLVs, with resting-state data from the “comfort” and “fatigue” conditions being used separately. A Hilbert transform was applied to electrodes *x* and *y* to extract the instantaneous phases at each time point [34]. The Equation (1) for calculating PLV is defined as follows:

(1) $$\mathrm{PLV}=\left|\frac{1}{N}\sum_{n=1}^{N}e^{j\left(\varphi_{x}\left(n\right)-\varphi_{y}\left(n\right)\right)}\right|$$

where $\varphi_{x}\left(n\right)-\varphi_{y}\left(n\right)$ is the instantaneous phase difference between electrodes *x* and *y* at each sample point in each epoch and *N* signifies the overall count of epochs. The PLV is averaged across all epochs to calculate the PLV for electrodes *x* and *y* within specific frequency bands. This calculation produces results spanning from 0 to 1, with 0 signifying the absence of phase synchronization and 1 representing complete phase synchronization for a specific electrode pair within a defined frequency band.

#### 2.5.2. Selection of Threshold Values

The choice of threshold *T* can significantly impact brain network construction and the analysis results [35]. However, based on previous expert studies, there is currently no definitive criterion or established methodology for selecting appropriate thresholds [36]. Achard and Bullmore discovered that sparse brain networks, consisting of the top 20% of all possible connections, typically maximize network topology efficiency [37]. Luo’s [38] research demonstrated that a sparsity level of 20% is optimal for capturing essential signal information and features. Lin et al. [39] selected a sparsity range of approximately 20% to study the brain network structure under conditions of fatigue. A sparsity level exceeding 20% results in a substantial computational burden, whereas a sparsity level below 20% risks overlooking significant edges. Therefore, we calculated the top 20% of PLV values for each participant across the alpha, theta, and delta frequency bands under comfort and fatigue conditions. Most participants had higher PLV values in the fatigue condition than in the comfort condition across all frequency bands. The mean PLV for the comfort and fatigue conditions ranged from 0.72 to 0.76 in steps of 0.01, establishing this range as the threshold intervals $T_{i}$ (i=1,2,3,4,5).

### 2.6. Graph Theory Analysis

Topological parameters are computed using MATLAB R2013b based on the brain network constructed by considering appropriate threshold intervals $T_{i}$ (i=1,2,3,4,5). The main focus is on various network properties. Node properties include betweenness centrality, nodal efficiency, and the clustering coefficient. Edge properties include characteristic path length.

#### 2.6.1. Betweenness Centrality

Betweenness centrality assesses the significance of a vertex based on its role in connecting other vertices via shortest paths. The betweenness centrality $c_{B}\left(j\right)$ of a given node *j* is calculated as the sum of the fractions of all pairs’ shortest paths that traverse node *j* [32]. The Equation (2) is defined as follows:

(2) $$c_{B}\left(j\right)=\sum \frac{\sigma_{st}\left(j\right)}{\sigma_{st\in V}}$$

$\sigma_{st}$ denotes the total count of shortest paths connecting nodes *s* and *t*, while $\sigma_{st}\left(j\right)$ signifies the quantity of those paths that traverse node *j* (excluding *s* and *t*). If *j* does not lie between *s* and *t*, then $\sigma_{st}\left(j\right)=0$; conversely, $\sigma_{st}=1$.

#### 2.6.2. Nodal Efficiency

Nodal efficiency quantifies a node’s capacity to disseminate information among other nodes within a network. A node exhibiting high nodal efficiency signifies a robust capability for information exchange with its peers, thereby qualifying it as a central hub. Equation (3), regarding nodal efficiency for specified node *j*, is defined as follows [40]:

(3) $$E_{\mathrm{nodal}\;}\left(j\right)=\frac{1}{N-1}\sum_{k\neq j\in V}\frac{1}{d_{j,k}}$$

$d_{j,k}$ denotes the shortest path length between nodes *j* and *k*. Nodal efficiency is measured on a scale of 0 to 1, reflecting the network’s capacity to relay and process information effectively among nodes. Essentially, the greater the nodal efficiency, the more adept the node is at transmitting information and integrating processes with its counterparts [41].

#### 2.6.3. Clustering Coefficient

The clustering coefficient serves as a pivotal metric, delineating the extent of closeness among nodes within a complex brain network. It can be divided into global and local clustering coefficients. The global clustering coefficient quantifies the average extent of clustering across all nodes within a network, serving as a measure to evaluate the network’s structural cohesion. The local clustering coefficient focuses on the connectivity between a single node and its neighbors. The ratio is determined by the number of edges incident to node *i* divided by the total number of edges that could potentially exist between its adjacent nodes. Equation (4) is defined as follows:

(4) $$C_{i}=\frac{2E_{i}}{k_{i}\left(k_{i}-1\right)}$$

$k_{i}$ signifies the number of neighboring nodes connected to a specific node *i* and $k_{i}\left(k_{i}-1\right)/2$ denotes the utmost number of edges that could potentially exist between these neighboring nodes. Equation (5) defines the global clustering coefficient:

(5) $$CC=\frac{1}{N}\sum_{i=1}^{N}c_{i}$$

where $c_{i}$ denotes the local clustering coefficient of node *i* and *N* signifies the total count of nodes [42].

#### 2.6.4. Characteristic Path Length

The characteristic path length is an important measure of the overall connectivity efficiency of a network. It is defined as the average length of the shortest path between all electrode pairs in a network. A reduced characteristic path length signifies a more efficient network for information transfer [24]. Equation (6) is defined as follows:

(6) $$L=\frac{1}{N(N-1)}\sum_{i,j\in N,i\neq j}d_{ij}$$

since this graph network is a weighted undirected graph, *N* denotes the quantity of electrodes and the edges symbolize the connections between electrode pairs. The weights of the edges reflect the strength of the connections between the electrode pairs. The Floyd–Warshall algorithm calculates the shortest path length between any two electrodes, excluding the distance from an electrode to itself [43]. When there is neither a direct nor an indirect connection between electrode pairs, the path length between them is deemed to be infinite. These infinite values are excluded when calculating the average characteristic path length. $d_{ij}$ represents the strength of the functional connections between the electrode pairs.

### 2.7. Statistical Tests

The topological properties of the participants’ brain networks under two distinct conditions—comfortable and fatigued—were statistically analyzed using SPSS software (SPSS 26.0 for Windows). First, a Shapiro–Wilk test was used to check if the data were normally distributed, and the data continuity was then validated. When the data exhibited normality, a paired *t*-test was utilized for analysis; conversely, the Wilcoxon signed-rank test was applied for data that did not meet the criteria for normal distribution.

## 3. Results

### 3.1. Brain Network Distribution Map Comparison

**Figure 4:**
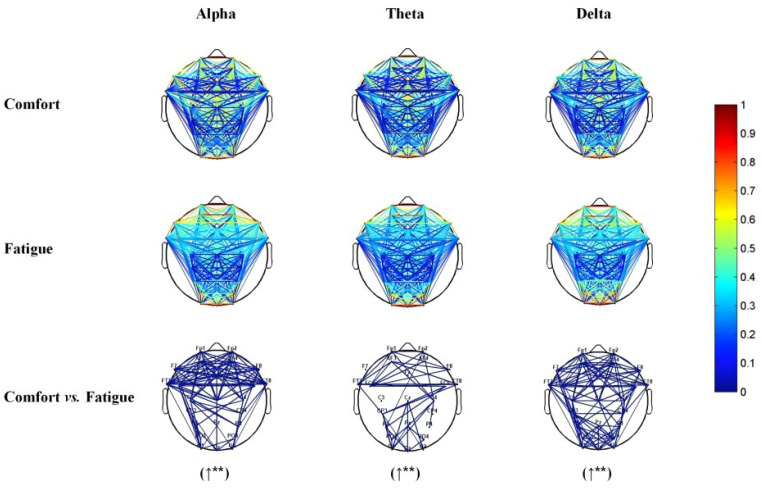
Average network distribution of two conditions of participants. Note: ↑ indicates that the mean PLV in the fatigue state is higher compared to the comfort state. **: $p_{\mathrm{mean}}<0.01$, where $p_{\mathrm{mean}}$ represents the mean of all significant electrode pairs.

Figure 4 and Table 1 present the average functional connectivity maps derived from PLV analysis in the alpha, theta and delta frequency bands separately. By computing the mean connectivity strength between each electrode pair in both comfort and fatigue states, a paired t-test, adjusted for a false discovery rate (FDR) to account for multiple comparisons, was employed to identify electrode pairs with significant differences. The significant electrode pairs, situated across various brain regions, were visualized within the alpha, theta, and delta frequency bands. Compared to the comfort condition, brain functional connectivity is significantly higher in the frontal, central, slightly parietal, and occipital regions in the alpha band during the fatigue condition ($p_{\mathrm{mean}}<0.01$); there is significantly higher functional brain connectivity in the frontal, right central, parietal, and occipital regions in the theta band ($p_{\mathrm{mean}}<0.01$); moreover, the functional brain connectivity across the entire brain regions were significantly enhanced in the delta band ($p_{\mathrm{mean}}<0.01$).

### 3.2. Results of the Graphical Comparison

**Figure 5:**
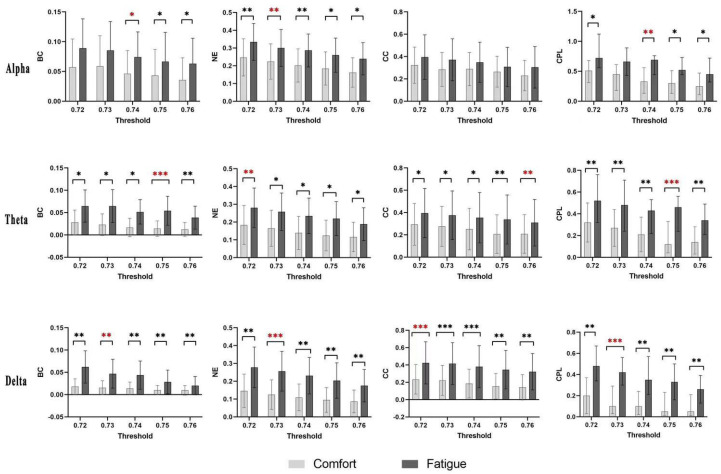
Comparative analysis of brain functional network metrics across the alpha, theta, and delta frequency bands among participants with varying thresholds. *: p<0.05, **: p<0.01, ***: p<0.001. Note: red signifies the threshold exhibiting the highest level of significance.

The main focus is to assess whether the mean values of betweenness centrality (BC), nodal efficiency (NE), the clustering coefficient (CC), and characteristic path length (CPL) are significantly different within the selected threshold interval. As shown in Figure 5, BC, NE, CC, and CPL values in the fatigue condition are generally higher than those in the comfort condition, especially around the threshold value of 0.75 in the alpha band. Significant differences between thresholds are more consistent in the theta band, where all network property values are higher in the fatigue state, especially between thresholds 0.72 to 0.75. The fatigue state also shows higher values for all properties in the delta band, with significant differences across multiple thresholds, especially between 0.72 and 0.75, highlighting a similar trend to the theta band. Analysis of the brain regions corresponding to significant differences between BC, NE, CC, and CPL in comfort and fatigue states at local scales facilitates an in-depth understanding of the practical significance of node and edge properties. Based on the outcomes presented in Table 2, Table 3 and Table 4, the thresholds exhibiting the most significant differences are selected for further analysis. Specifically, BC exhibits the most significant performance when the threshold values are 0.74 for the alpha band (p<0.05), 0.75 for the theta band (p<0.001), and 0.73 for the delta band (p<0.001). Similarly, NE demonstrates its most prominent performance with threshold values of 0.73 in the alpha band (p<0.01), 0.72 in the theta band (p<0.01), and 0.73 in the delta band (p<0.001).The CC achieves its peak performance with threshold values of 0.76 in the theta band (p<0.01) and 0.72 in the delta band (p<0.001). Lastly, CPL also exhibits significant performance with threshold values of 0.74 in the alpha band (p<0.05), 0.75 in the theta band (p<0.001), and 0.73 in the delta band (p<0.001).

### 3.3. Brain Network Node Properties Analysis

**Figure 6:**
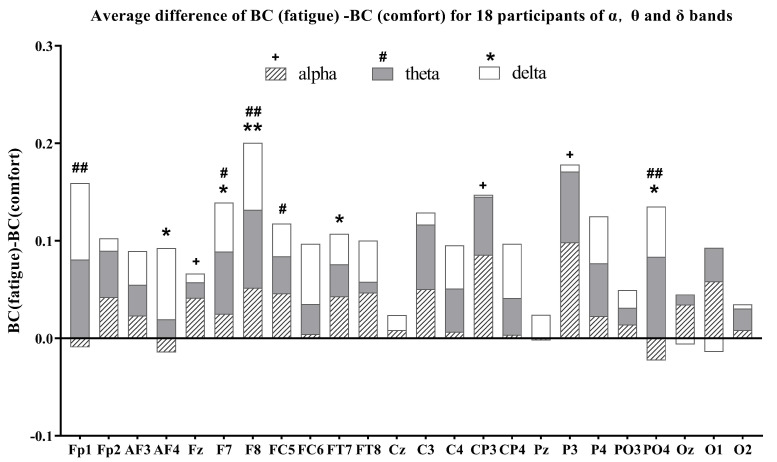
Average difference of BC (fatigue)-BC (comfort) for all participants of the alpha, theta, and delta bands. ^+^, ^#^, *: p<0.05; ^##^, **: p<0.01.

**Figure 7:**
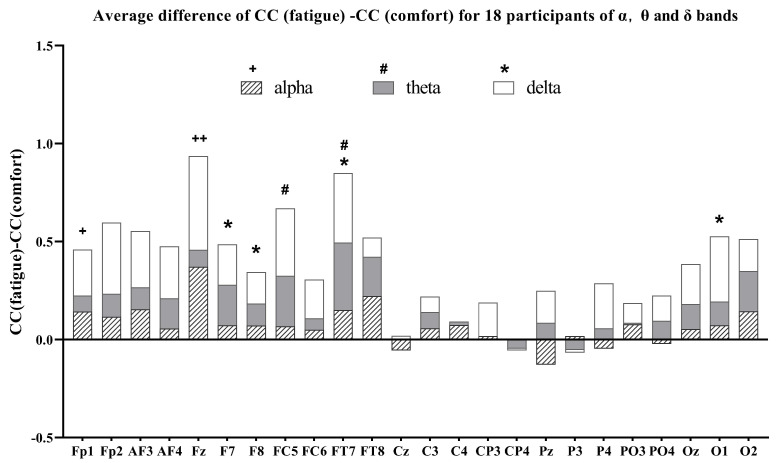
Average difference of CC (fatigue)-CC (comfort) for all participants of the alpha, theta, and delta bands. ^+^, ^#^, *: p<0.05; ^++^: p<0.01.

**Figure 8:**
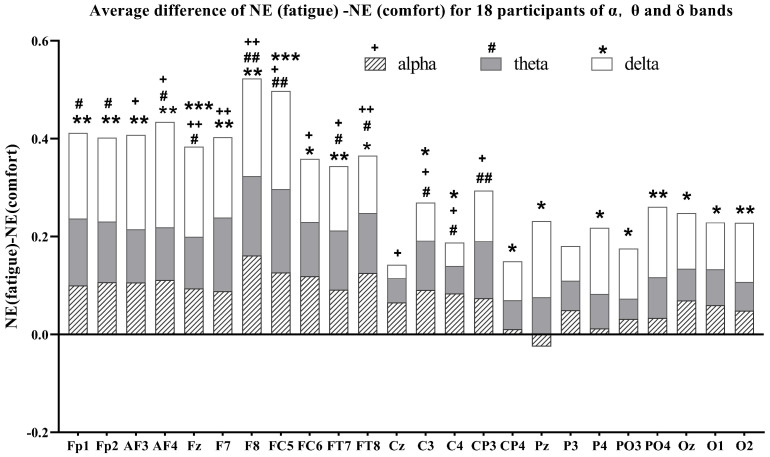
Average difference of NE (fatigue)-NE (comfort) for all participants of the alpha, theta, and delta bands. ^+^, ^#^, *: p<0.05; ^++^, ^##^, **: p<0.01; ***: p<0.001.

As mentioned above, the average difference between the fatigue state (after watching) and the comfort state (before watching) for node properties in 24 channels —betweenness centrality (BC), nodal efficiency (NE), and the cluster coefficient (CC) —was computed across the three EEG frequency bands to identify significant brain regions. The average difference of the alpha, theta, and delta band node properties for the 18 participants are given in Figure 6, Figure 7 and Figure 8, respectively.

In the alpha band for the BC node property, the BC (fatigue) values are greater than the BC (comfort) values for all channels except Fp1, AF4, and PO4. This means the BC value increases after watching. Statistical analysis revealed significant differences for Fz, CP3, and P3 channels before and after watching. In the alpha band of CC node properties, all channels in the frontal, temporal, and occipital lobes show higher cluster coefficients pre-watching compared to post-watching. This is also true for all channels in the central and parietal lobes except for Cz, Pz, P4, and PO4. Cz, Pz, and P4 are the channels with higher values of clustering coefficients pre-watching compared to post-watching. Significant differences in CCs are concentrated in the Fp1 and Fz channels. In the alpha band of NE node properties, the nodal efficiency values after watching are greater than before watching in all channels except Pz, where the opposite is true for Pz. Significant differences in the NE are observed in more channels, i.e., AF3, AF4, Fz, F8, FC5, FC6, FT7, FT8, Cz, C3, C4, and CP3.

In the theta band of Figure 6, all channels exhibit higher BC values post-watching compared to pre-watching, with significant differences being observed in Fp1, F7, F8, FC5, and PO4. In the theta band of Figure 7, almost all channels exhibit larger post-watching CC values than pre-watching ones, except for CP4 and P3, where the situation is reversed. Notable differences are evident in the CC values between FC5 and FT7. In the theta band of Figure 8, the NE values after watching are also higher than before watching for all channels. For some meaningful channels, i.e., Fp1, Fp2, AF4, Fz, F7, F8, FC5, FT7, FT8, C3, C4, and CP3, the nodal efficiencies after watching are significantly larger than those before watching.

In the delta bands for the BC node property, BC (fatigue) is positive compared to BC (comfort) in almost all channels except Oz and O1. The same is true for the CC node property in all channels except CP4 and P3. For the meaningful channels, i.e., AF4, F7, F8, FT7, and PO4, the BC value after watching is significantly greater than the BC value before watching. For the meaningful channels, i.e., F7, F8, FT7, and O1, the CC after watching is significantly larger than before watching. In the delta band of NE node properties, the NE values after watching are higher than before watching for all channels. Notably, all channels in the frontal, temporal, and occipital lobes show a significantly higher NE value after watching. This is also the case for all channels in the central and parietal lobes, except for Cz and P3.

Overall, a statistical analysis of node properties was conducted before watching (comfort) and after watching (fatigue) across different frequency bands. Specifically, in the alpha band, the significance of the BC is located in the frontal, central, and parietal regions; in the theta band, these differences are observed in the frontal and parietal regions; and, in the delta band, these differences are observed in the frontal, temporal, and parietal regions. The primary focus of these significant differences in BC is on the frontal, parietal, and, to a lesser extent, temporal and central regions. For the CC, significant changes in the alpha band are evident in the frontal region; in the theta band, they are presented in both the frontal and temporal regions; and, in the delta band, they are located in the frontal, temporal, and occipital regions. Significant differences regarding the CC are concentrated in the frontal region, with a mild concentration in the temporal and occipital regions. In terms of nodal efficiency, significant changes in the alpha band are concentrated in the frontal, temporal, and central regions; significant changes in the theta band are located in the frontal, temporal, and central regions; and significant changes in the delta band are located in the frontal, temporal, central, parietal and occipital regions. The significant differences in NE are primarily concentrated in the frontal, temporal, and central regions, with slight differences occurring in the parietal and occipital regions.

### 3.4. Brain Network Edge Properties Analysis

**Figure 9:**
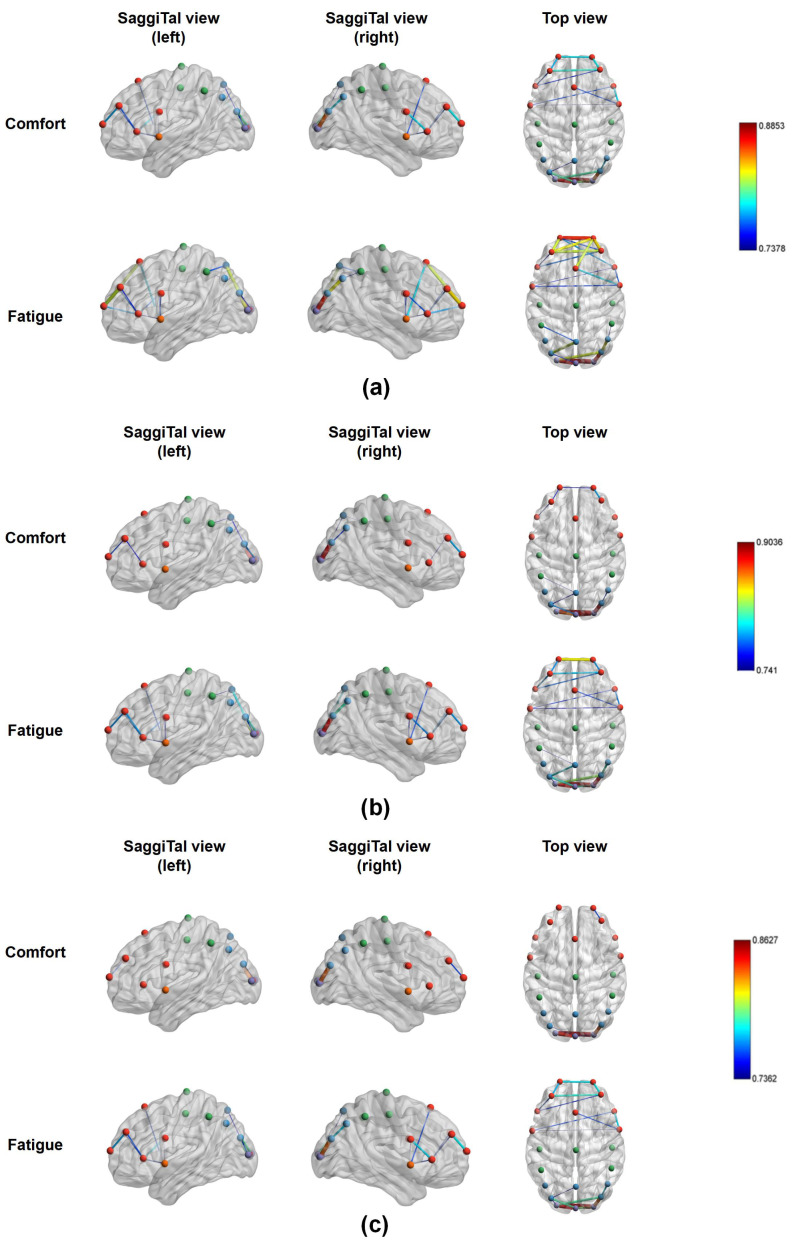
3D Modular Brain Networks. (**a**) represents the alpha band. (**b**) represents the theta band. (**c**) represents the delta band. Each band corresponds to both comfort and fatigue states. Different nodes have different colors, and the color of the edges changes according to the colors of the nodes. The edge thickness signifies the strength of the connection between nodes. A thicker edge indicates a stronger relationship. The brain networks were visualized using the BrainNet Viewer (http://www.nitrc.org/projects/bnv/, accessed on 15 December 2023) [45].

The study is extended to include edge properties to further investigate brain regions associated with visual fatigue. Specifically, the focus is on the characteristic path length (CPL) of network edges, which reflects variations in brain network organization between comfort and fatigue states. Figure 9 visually represents the modular brain functional networks corresponding to the alpha, theta, and delta bands, highlighting the differences observed between the comfort and fatigue states. The analysis reveals that the network in a state of comfort exhibits greater organization, characterized by fewer interconnections between brain regions, maintaining more efficient pathways for information exchange. In contrast, connections between brain regions show a marked increase during the fatigue state, resulting in more disorganized information interactions. In addition, the higher characteristic path lengths observed in the fatigue state suggest an increase in the shortest paths connecting various brain regions, which in turn diminishes the parallel efficiency of information transmission [44].

## 4. Discussion

Many previous EEG-based stereo-visual fatigue experiments [21,46,47] have used 2D pictures with different static parallaxes as visual fatigue materials. However, studies based on conventional stereoscopic displays may be less convenient for real-world applications. In this study, a stereo-depth motion experimental paradigm was designed to induce visual fatigue, enabling researchers to simulate environments where the virtual and real worlds merge, providing a high degree of immersion and interactivity. However, participants in the experiment were required to observe the corresponding scenes continuously, which entailed certain cognitive processes such as visual attention, depth perception, and motion tracking. Gumilar et al. discovered that collaborative tasks enhance inter-brain connectivity [48]. Although there are various connectivity metrics, such as spectral coherence or Granger causality, the phase locking value (PLV) is widely used in the field of virtual reality (VR) [25,49]. Compared to those measures, a PLV may offer a more direct indication of synchronous activity between brain regions, making it particularly suitable for analyzing dynamic interactions in immersive environments [48].

Based on above comprehensive analysis, it has been observed that the dissemination of node characteristics across various frequency bands is extensive, notably featuring a pre-eminent frontal region. The occipital lobe serves as the center of the visual cortex, directly receiving information from the retina and the frontal lobe, making it more responsible for stereo and spatial information [23,50]. The flow of information permeates through multiple brain regions. Specifically, these characteristics are primarily located in the frontal and central regions, with slight involvement of the temporal and parietal regions in the alpha band. In the theta band, these characteristics are primarily located in frontal and temporal regions, with slight involvement of the central and parietal regions. In the delta band, the characteristics are located primarily in the frontal, temporal, parietal, and occipital regions, with slight involvement of the central region. The significant differences in node properties located across multiple brain regions are consistent with research on brain networks during states of visual fatigue [20].

As the level of visual fatigue increases, the BC, CC, and NE in different frequency bands increase significantly in the fatigue state, with alterations in NE nearly encompassing the entire brain region (Figure 8). The improvements in global efficiency are often associated with an overactive functional integration [40]. In a state of fatigue, the transfer of information between brain regions presents excessive cognitive load, leading to an increase in global efficiency metrics. Nevertheless, this rise in efficiency is also accompanied by a higher actual cost of information transmission, reflecting strives to maintain normal cognitive functions under fatigue, which is consistent with research on brain networks in cognitive states [25,51]. Yue and Wang found that stereoscopic visual fatigue may be associated with attention and parallax processing [21]. Given that theta oscillations in the EEG are associated with memory, the increase in the CC in the theta band, located in the frontal and temporal region, means an elevated local efficiency of information transmission [51], resulting in greater cognitive process during fatigue (Figure 7). Similarly, the increase in BC in the theta band suggests a significantly functional reorganization in the frontal and parietal region (Figure 6), implying greater cognitive demand during fatigue. Zhao et al. [52] found that the CC increased significantly in the alpha and delta bands in terms of driving-induced mental fatigue. The increase in the CC means that the connectivity between neighboring nodes has been strengthened [24]. We think that the increase in the CC in the frontal region of the alpha and delta bands means that the induction of stereo-visual fatigue caused participants to encounter difficulties in focusing their visual perceptions and maintaining concentration to complete the viewing task (Figure 7). The information flow of BC in the alpha and delta band primarily concentrates on the frontal, temporal, central and parietal regions, suggesting a greater involvement in visual processing (Figure 6).

**Figure 10:**
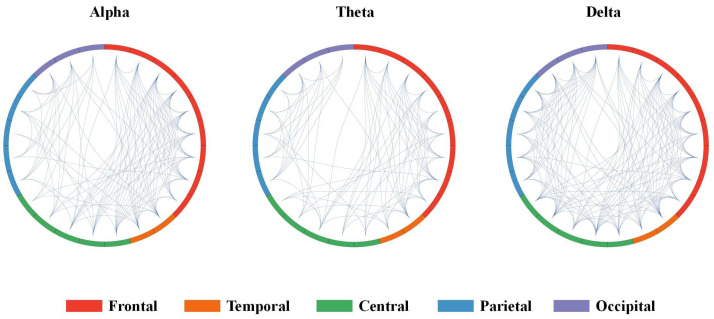
The significance of alpha, theta, and delta in the comfort and fatigue states was assessed using a one-tailed test.

Compared to the comfort state, the edge characteristics in the alpha, theta, and delta frequency bands increase significantly over specific threshold intervals, with the most significant pronounced increases being seen in the theta and delta frequency bands (Figure 5). To specifically verify the significant differences in the fatigue state, a one-tailed test was employed (Figure 10). The results suggests an increase in the number of long-distance connections across the three frequency bands, resulting in disrupted effective interactions across brain regions [52]. The global connectivity patterns gradually transition from an ordered to a less efficient configuration [53], which reflects a loss of synaptic transmission efficiency on a global level. The elevated characteristic path lengths detected during the fatigue state disrupted the effective communication between brain regions, aligning with previous research findings [28,54].

In summary, the validation of these brain network properties in the alpha, theta, and delta frequency bands further supports the notion that it is indeed feasible to monitor visual fatigue through EEG functional connectivity within MR-based work environments. The widespread distribution of node and edge characteristics across different frequency bands reflects the global impact of fatigue on functional connectivity throughout the brain. We have observed that significant differences are primarily distributed in the frontal regions and engage in extensive interactions with the information flow from other brain areas. Our results indicate that topological characteristics in the delta and theta frequency bands were prominent in the transition from comfort to fatigue, which is consistent with previous studies [55]. These topological characteristic can be applied to fields with high cognitive demands, such as medicine and industry, to monitor fatigue states. However, the participants enrolled in this study were generally young adults. In future work, we will concentrate on analyzing the brain network structure in different age groups under MR stereo vision to further explore the underlying neural mechanisms.

## 5. Conclusions

In this paper, the EEG-based brain network characterization of stereo-visual fatigue in mixed-reality environments was investigated. By using a stereoscopic depth motion experimental paradigm as a visual stimulus, brain networks were constructed based on phase-locking values (PLVs). The mean PLV during the fatigue state is higher than that in the comfortable state across the alpha, theta, and delta frequency bands. The topological properties of these networks, including betweenness centrality (BC), node efficiency (NE), clustering coefficient (CC), and characteristic path length (CPL), were calculated to analyze brain network organization under comfort and fatigue states. The analysis results showed significant differences in the topological properties on a global level. For details, the significant difference of node properties (BC, NE and CC) are prominently distributed in the frontal region, with additional involvement of the temporal, central, parietal, and occipital regions. In addition, the edge property (CPL) significantly increase during the fatigue state across the alpha, theta and delta frequency bands. In our study, these topological properties can serve as valid indicators of comfort and fatigue states in MR content.

## Figures and Tables

**Table 1:** Specific information about the average network distribution map.

Band	PLV (Comfort/Fatigue) Mean (SD)	FDR-Corrected *p*-Value Mean (SD)
Alpha	0.51 (0.14)/0.53 (0.12)	0.006 (0.005)
Theta	0.50 (0.13)/0.52 (0.12)	0.003 (0.002)
Delta	0.50 (0.11)/0.54 (0.10)	0.006 (0.005)

**Table 2:** Statistical analysis of brain network properties in the alpha band across five thresholds.

Property	Threshold	Alpha (Comfort/Fatigue) Mean (SD) or Median (Q1∼Q3)		T-Statistic or Z-Statistic	*p*-Value
BC	0.72	0.04 (0.03∼0.07)	0.07(0.05∼0.13)	−1.895	0.058
	0.73	0.03 (0.02∼0.06)	0.06 (0.04∼0.10)	−1.328	0.184
	**0.74**	0.03 (0.01∼0.04)	0.06 (0.04∼0.09)	−2.526	**0.012**
	0.75	0.03 (0.01∼0.04)	0.05 (0.03∼0.08)	−2.156	**0.031**
	0.76	0.03 (0.01∼0.04)	0.04 (0.03∼0.06)	−2.178	**0.029**
NE	0.72	0.25 (0.14)	0.28 (0.22∼0.44)	−2.722	**0.006**
	**0.73**	0.2 3 (0.15)	0.31 (0.16)	−3.126	**0.006**
	0.74	0.20 (0.14)	0.29 (0.16)	−2.935	**0.009**
	0.75	0.15 (0.08∼0.26)	0.27 (0.15)	−2.504	**0.012**
	0.76	0.11 (0.07∼0.25)	0.24 (0.15)	−2.373	**0.018**
CC	0.72	0.32 (0.18)	0.40 (0.19)	−1.694	0.108
	0.73	0.29 (0.19)	0.37 (0.21)	−1.754	0.097
	0.74	0.29 (0.19)	0.35 (0.20)	−1.460	0.163
	0.75	0.26 (0.18)	0.32 (0.19)	−1.566	0.136
	**0.76**	0.23 (0.18)	0.30 (0.20)	−1.955	0.067
CPL	0.72	0.51 (0.31∼0.68)	0.72(0.56∼1.12)	−2.330	**0.020**
	0.73	0.45 (0.18∼0.61)	0.66 (0.43∼0.89)	−1.677	0.094
	**0.74**	0.33 (0.13∼0.56)	0.69 (0.44∼0.76)	−2.817	**0.005**
	0.75	0.30 (0.13∼0.51)	0.52 (0.44∼0.73)	−2.461	**0.014**
	0.76	0.25 (0.11∼0.47)	0.45 (0.32∼0.72)	−2.091	**0.037**

Bolded *p*-values correspond to brain network properties that are significant in Figure 5 under the alpha band; bolded thresholds are chosen for subsequent analysis.

**Table 3:** Statistical analysis of brain network properties in the Theta band across five thresholds.

Property	Threshold	Theta (Comfort/Fatigue) Mean (SD) or Median (Q1∼Q3)		T-Statistic or Z-Statistic	*p*-Value
BC	0.72	0.03 (0.02)	0.04 (0.03∼0.08)	−2.417	**0.016**
	0.73	0.02 (0.02)	0.04 (0.02∼0.08)	−2.417	**0.016**
	0.74	0.01 (0.00∼0.02)	0.04 (0.02∼0.05)	−2.504	**0.012**
	**0.75**	0.01 (0.01)	0.04 (0.02∼0.06)	−3.549	**0.000**
	0.76	0.01 (0.00∼0.02)	0.03 (0.02∼0.05)	−2.911	**0.004**
NE	**0.72**	0.18 (0.11)	0.28 (0.13)	−3.214	**0.005**
	0.73	0.13 (0.07∼0.24)	0.26 (0.12)	−2.504	**0.012**
	0.74	0.10 (0.05∼0.23)	0.23 (0.11)	−2.570	**0.010**
	0.75	0.09 (0.04∼0.21)	0.19 (0.14∼0.31)	−2.461	**0.014**
	0.76	0.09 (0.03∼0.19)	0.17 (0.12∼0.24)	−2.243	**0.025**
CC	0.72	0.30 (0.17)	0.40 (0.14)	−2.275	**0.036**
	0.73	0.28 (0.18)	0.38 (0.12)	−2.309	**0.034**
	0.74	0.25 (0.16)	0.35 (0.11)	−2.481	**0.024**
	0.75	0.20 (0.17)	0.34 (0.11)	−3.217	**0.005**
	**0.76**	0.18 (0.14)	0.30 (0.13)	−3.227	**0.005**
CPL	0.72	0.32 (0.18)	0.52 (0.32∼0.76)	−2.983	**0.003**
	0.73	0.27 (0.17)	0.48 (0.24∼0.71)	−2.765	**0.006**
	0.74	0.21 (0.16)	0.43 (0.22∼0.53)	−3.463	**0.001**
	**0.75**	0.12 (0.04∼0.33)	0.46 (0.24∼0.56)	−3.680	**0.000**
	0.76	0.14 (0.03∼0.28)	0.34 (0.21∼0.49)	−2.940	**0.003**

Bolded *p*-values correspond to brain network properties that are significant in Figure 5 under the theta band; bolded thresholds are chosen for subsequent analysis.

**Table 4:** Statistical analysis of brain network properties in the delta band across five thresholds.

Property	Threshold	Delta (Comfort/Fatigue) Mean (SD) or Median (Q1∼Q3)		T-Statistic or Z-Statistic	*p*-Value
BC	0.72	0.02 (0.01)	0.04 (0.02∼0.06)	−3.245	**0.001**
	**0.73**	0.01 (0.00∼0.03)	0.03 (0.02∼0.05)	−3.375	**0.001**
	0.74	0.01 (0.00∼0.02)	0.03 (0.02∼0.05)	−3.245	**0.001**
	0.75	0.00 (0.00∼0.02)	0.03 (0.02)	−3.288	**0.001**
	0.76	0.00 (0.00∼0.01)	0.02 (0.01)	−2.505	**0.012**
NE	0.72	0.10 (0.05∼0.20)	0.28(0.12)	−3.375	**0.001**
	**0.73**	0.08 (0.03∼0.17)	0.22 (0.17∼0.33)	−3.506	**0.000**
	0.74	0.05 (0.02∼0.14)	0.19 (0.15∼0.31)	−3.462	**0.001**
	0.75	0.04 (0.02∼0.13)	0.17 (0.14∼0.26)	−3.207	**0.002**
	0.76	0.04 (0.02∼0.12)	0.14 (0.12∼0.21)	−2.853	**0.004**
CC	**0.72**	0.22 (0.17)	0.43(0.12)	−6.202	**0.000**
	0.73	0.21 (0.17)	0.42 (0.12)	−5.734	**0.000**
	0.74	0.17 (0.15)	0.38 (0.12)	−5.395	**0.000**
	0.75	0.12 (0.00∼0.25)	0.35 (0.13)	−3.432	**0.001**
	0.76	0.11 (0.00∼0.23)	0.32 (0.13)	−3.148	**0.002**
CPL	0.72	0.20 (0.17)	0.48 (0.34∼0.67)	−3.332	**0.001**
	**0.73**	0.10 (0.03∼0.29)	0.42 (0.30∼0.56)	−3.636	**0.000**
	0.74	0.10 (0.02∼0.24)	0.35 (0.21∼0.57)	−3.462	**0.001**
	0.75	0.05 (0.01∼0.23)	0.33 (0.17)	−3.397	**0.001**
	0.76	0.05 (0.01∼0.21)	0.26 (0.13)	−2.765	**0.006**

Bolded *p*-values correspond to brain network properties that are significant in Figure 5 under the delta band; bolded thresholds are chosen for subsequent analysis.

## Data Availability

In this study, publicly available data sets were analyzed. This data can be assessed via the following link: https://github.com/taochunguang2022/data (accessed on 16 July 2024).

## References

[1] Hu H.z., Feng X.b., Shao Z.w., Xie M., Xu S., Wu X.h., Ye Z.w. (2019). Application and prospect of mixed reality technology in medical field. Curr. Med. Sci..

[2] Huang Y., Cheng X., Chan U., Zheng L., Hu Y., Sun Y., Lai P., Dai J., Yang X. (2022). Virtual reality approach for orthodontic education at School of Stomatology, Jinan University. J. Dent. Educ..

[3] Banquiero M., Valdeolivas G., Ramón D., Juan M.C. (2024). A color Passthrough mixed reality application for learning piano. Virtual Real..

[4] Yao W., Wang L., Liu D. (2024). Augmented Reality-Based Language and Math Learning Applications for Preschool Children Education. Universal Access in the Information Society.

[5] Han J., Bae S.H., Suk H.J. (2017). Visual Discomfort and Visual Fatigue: Comparing Head-Mounted Display and Smartphones: Comparing Head-Mounted Display and Smartphones. J. Ergon. Soc. Korea.

[6] Hirota M., Kanda H., Endo T., Miyoshi T., Miyagawa S., Hirohara Y., Yamaguchi T., Saika M., Morimoto T., Fujikado T. (2019). Comparison of visual fatigue caused by head-mounted display for virtual reality and two-dimensional display using objective and subjective evaluation. Ergonomics.

[7] Hua H. (2017). Enabling focus cues in head-mounted displays. Proc. IEEE.

[8] Fan L., Wang J., Li Q., Song Z., Dong J., Bao F., Wang X. (2023). Eye movement characteristics and visual fatigue assessment of virtual reality games with different interaction modes. Front. Neurosci..

[9] Wang X., Liu L., Hu X., Wu Y., Liu Y., Ni B., Ke B. (2023). Comparison of changes in visual fatigue and ocular surface after 3D and 2D viewing with augmented reality glasses. Displays.

[10] Cometti C., Païzis C., Casteleira A., Pons G., Babault N. (2018). Effects of mixed reality head-mounted glasses during 90 minutes of mental and manual tasks on cognitive and physiological functions. PeerJ.

[11] Chen C., Li K., Wu Q., Wang H., Qian Z., Sudlow G. (2013). EEG-based detection and evaluation of fatigue caused by watching 3DTV. Displays.

[12] Zhang C., Yu X., Yang Y., Xu L. (2014). Phase synchronization and spectral coherence analysis of EEG activity during mental fatigue. Clin. EEG Neurosci..

[13] Zhao X., Wu J., Peng H., Beheshti A., Monaghan J.J., McAlpine D., Hernandez-Perez H., Dras M., Dai Q., Li Y. (2022). Deep reinforcement learning guided graph neural networks for brain network analysis. Neural Netw..

[14] Guo M., Yue K., Hu H., Lu K., Han Y., Chen S., Liu Y. (2022). Neural research on depth perception and stereoscopic visual fatigue in virtual reality. Brain Sci..

[15] Lee C.C., Chiang H.S., Hsiao M.H. (2021). Effects of screen size and visual presentation on visual fatigue based on regional brain wave activity. J. Supercomput..

[16] Zou B., Liu Y., Guo M., Wang Y. (2015). EEG-based assessment of stereoscopic 3D visual fatigue caused by vergence-accommodation conflict. J. Disp. Technol..

[17] Kim H.K., Park J., Choi Y., Choe M. (2018). Virtual reality sickness questionnaire (VRSQ): Motion sickness measurement index in a virtual reality environment. Appl. Ergon..

[18] Di Gregorio F., Battaglia S. (2023). Advances in EEG-based functional connectivity approaches to the study of the central nervous system in health and disease. Adv. Clin. Exp. Med..

[19] Wang X., Yao L., Zhao Y., Xing L., Qian Z., Li W., Yang Y. (2018). Effects of disparity on visual discomfort caused by short-term stereoscopic viewing based on electroencephalograph analysis. BioMed. Eng. OnLine.

[20] Chen C., Wang J., Li K., Liu Y., Chen X. (2015). Visual fatigue caused by watching 3DTV: An fMRI study. BioMed. Eng. Online.

[21] Yue K., Wang D. (2021). Investigating the neural activity of various 3D visual fatigue degrees using depth-related visual evoked potentials. J. Soc. Inf. Disp..

[22] Krokos E., Varshney A. (2022). Quantifying VR cybersickness using EEG. Virtual Real..

[23] Zhang T., Guo M., Wang L., Li M. (2022). Brain fatigue analysis from virtual reality visual stimulation based on granger causality. Displays.

[24] Han C., Sun X., Yang Y., Che Y., Qin Y. (2019). Brain complex network characteristic analysis of fatigue during simulated driving based on electroencephalogram signals. Entropy.

[25] Yu M., Li Y., Tian F. (2021). Responses of functional brain networks while watching 2D and 3D videos: An EEG study. Biomed. Signal Process. Control.

[26] Kar S., Routray A. (2012). Effect of sleep deprivation on functional connectivity of EEG channels. IEEE Trans. Syst. Man Cybern. Syst..

[27] Tian P., Xu G., Han C., Zheng X., Zhang K., Du C., Wei F., Zhang S. (2022). Effects of paradigm color and screen brightness on visual fatigue in light environment of night based on eye tracker and EEG acquisition equipment. Sensors.

[28] Chen J., Wang S., He E., Wang H., Wang L. (2023). The architecture of functional brain network modulated by driving during adverse weather conditions. Cogn. Neurodyn..

[29] Chen N., Zhao M., Gao K., Zhao J. (2020). The physiological experimental study on the effect of different color of safety signs on a virtual subway fire escape—An exploratory case study of zijing mountain subway station. Int. J. Environ. Res. Public Health.

[30] Delorme A., Makeig S. (2004). EEGLAB: An open source toolbox for analysis of single-trial EEG dynamics including independent component analysis. J. Neurosci. Methods.

[31] Pion-Tonachini L., Kreutz-Delgado K., Makeig S. (2019). ICLabel: An automated electroencephalographic independent component classifier, dataset, and website. NeuroImage.

[32] Yu M., Xiao S., Hua M., Wang H., Chen X., Tian F., Li Y. (2022). EEG-based emotion recognition in an immersive virtual reality environment: From local activity to brain network features. Biomed. Signal Process. Control.

[33] Shao G., Xu G., Huo C., Nie Z., Zhang Y., Yi L., Wang D., Shao Z., Weng S., Sun J. (2024). Effect of the VR-guided grasping task on the brain functional network. Biomed. Opt. Express.

[34] Brunner C., Scherer R., Graimann B., Supp G., Pfurtscheller G. (2006). Online control of a brain-computer interface using phase synchronization. IEEE Trans. Biomed. Eng..

[35] Jalili M. (2016). Functional Brain Networks: Does the Choice of Dependency Estimator and Binarization Method Matter?. Sci. Rep..

[36] Niu X., Chi P., Song J., Pang Y., Wu Q., Liu Y., Chi A. (2022). Effects of sleep deprivation on functional connectivity of brain regions after high-intensity exercise in adolescents. Sustainability.

[37] Achard S., Bullmore E. (2007). Efficiency and cost of economical brain functional networks. PLoS Comput. Biol..

[38] Luo H., Qiu T., Liu C., Huang P. (2019). Research on fatigue driving detection using forehead EEG based on adaptive multi-scale entropy. Biomed. Signal Process. Control.

[39] Lin Z., Qiu T., Liu P., Zhang L., Zhang S., Mu Z. (2021). Fatigue driving recognition based on deep learning and graph neural network. Biomed. Signal Process. Control.

[40] Ma X., Jiang G., Fu S., Fang J., Wu Y., Liu M., Xu G., Wang T. (2018). Enhanced network efficiency of functional brain networks in primary insomnia patients. Front. Psychiatry.

[41] Stanley M.L., Simpson S.L., Dagenbach D., Lyday R.G., Burdette J.H., Laurienti P.J. (2015). Changes in Brain Network Efficiency and Working Memory Performance in Aging. PLoS ONE.

[42] Zheng R., Wang Z., He Y., Zhang J. (2022). EEG-based brain functional connectivity representation using amplitude locking value for fatigue-driving recognition. Cogn. Neurodyn..

[43] Toroslu I.H. (2023). The Floyd-Warshall all-pairs shortest paths algorithm for disconnected and very sparse graphs. Softw. Pract. Exp..

[44] Wu J., Zhang J., Ding X., Li R., Zhou C. (2013). The effects of music on brain functional networks: A network analysis. Neuroscience.

[45] Xia M., Wang J., Viewer Y.H.B. (2013). A network visualization tool for human brain connectomics. PLoS ONE.

[46] Wang X., Yao L., Qian Z., Xing L., Li W., Yang Y. (2018). Excessive Crossed Disparity Detection by Visual Evoked Potentials to Reduce Visual Discomfort in 3D Viewing. Comput. Intell. Neurosci..

[47] Zheng Y., Zhao X., Yao L. (2019). The assessment of the visual discomfort caused by vergence-accommodation conflicts based on EEG. J. Soc. Inf. Disp..

[48] Gumilar I., Sareen E., Bell R., Stone A., Hayati A., Mao J., Barde A., Gupta A., Dey A., Lee G. (2021). A comparative study on inter-brain synchrony in real and virtual environments using hyperscanning. Comput. Graph..

[49] Qu J., Cui L., Guo W., Ren X., Bu L. (2022). The effects of a virtual reality rehabilitation task on elderly subjects: An experimental study using multimodal data. IEEE Trans. Neural Syst. Rehabil. Eng..

[50] Kim J.H., Cho J.Y. (2018). Characteristics of the process of visual attention during spatial depth perception. Sci. Emot. Sensib..

[51] Kitzbichler M.G., Henson R.N., Smith M.L., Nathan P.J., Bullmore E.T. (2011). Cognitive effort drives workspace configuration of human brain functional networks. J. Neurosci..

[52] Zhao C., Zhao M., Yang Y., Gao J., Rao N., Lin P. (2016). The reorganization of human brain networks modulated by driving mental fatigue. IEEE J. Biomed. Health Inform..

[53] Sun Y., Lim J., Kwok K., Bezerianos A. (2014). Functional cortical connectivity analysis of mental fatigue unmasks hemispheric asymmetry and changes in small-world networks. Brain Cogn..

[54] Chen J., Wang S., He E., Wang H., Wang L. (2022). Two-dimensional phase lag index image representation of electroencephalography for automated recognition of driver fatigue using convolutional neural network. Expert Syst. Appl..

[55] Chen J., Wang H., Hua C., Wang Q., Liu C. (2018). Graph analysis of functional brain network topology using minimum spanning tree in driver drowsiness. Cogn. Neurodyn..

